# Efficacy and safety of a thermosensitive hydrogel for endoscopic submucosal dissection: An in vivo swine study

**DOI:** 10.1371/journal.pone.0260458

**Published:** 2021-12-09

**Authors:** Han Jo Jeon, Hyuk Soon Choi, Eun Ju Bang, Kang Won Lee, Sang Hyun Kim, Jae Min Lee, Eun Sun Kim, Bora Keum, Yoon Tae Jeen, Hong Sik Lee, Hoon Jai Chun, Seung Jeong, Jong Hyuk Kim

**Affiliations:** 1 Division of Gastroenterology and Hepatology, Department of Internal Medicine, Korea University College of Medicine, Seoul, Republic of Korea; 2 Department of Biosystems & Biomaterials Science and Engineering, Seoul National University, Seoul, Republic of Korea; 3 Department of Veterinary Clinical Sciences, College of Veterinary Medicine, University of Minnesota, St. Paul, MN, United States of America; 4 Masonic Cancer Center, University of Minnesota, Minneapolis, MN, United States of America; Brandeis University, UNITED STATES

## Abstract

Injectable thermo-sensitive chitosan hydrogels have recently been developed for the use of submucosal fluids in endoscopic submucosal dissections (ESD). This study aimed to investigate the efficacy and safety of chitosan hydrogels during ESD. Submucosal fluids were administered as follows: 0.9% normal saline (NS), 0.4% hyaluronic acid (HA) and chitosan/β-glycerophosphate (CS/GP) hydrogel. Each solution was administered twice into the stomach and colon of a pig, with a total of 72 ESD procedures performed on 12 pigs. The injected volume and procedure-related parameters were recorded and analyzed. ESDs that created ulcers after 7 days were histologically compared. All ESD specimens were resected en bloc. The total injected volumes during ESD of the stomach (NS, 16.09±3.27 vs. HA, 11.17±2.32 vs. CS/GP, 9.44±2.33; p<0.001) and colon (NS, 9.17±1.80 vs. HA, 6.67±1.50 vs. CS/GP, 6.75±1.57; p = 0.001) were significantly different. Hydrogel showed significant differences from normal saline in terms of fluid power (mm^2^/vol; NS, 35.70±9.00 vs. CS/GP 57.48±20.77; p = 0.001) and consumption rate (vol/min; NS, 2.59±0.86 vs. CS/GP, 1.62±0.65; p = 0.013) in the stomach. Histological examination revealed preserved muscularis propria, although the chitosan hydrogel resulted in a partial inflammatory response, with a hypertrophied submucosal layer. Chitosan hydrogel was found to be superior to normal saline, with an efficacy similar to that of hyaluronic acid. Nonetheless, long-term histological changes should be evaluated before clinical implementation.

## Introduction

Endoscopic submucosal dissection (ESD) is a well-established technique for eliminating mucosa- or submucosa-confined superficial neoplastic lesions from the muscularis propria layer [1]. Studies have shown that, compared with radical surgery, ESD, known to have similar oncological efficacy and fewer complications in early cancer has no difference in overall survival [2–4]. ESD has made possible en bloc R0 resection in T1 stage tumors, which would have been impossible without the assistance of submucosal solution. The widespread practice of ESD consequently popularized the use of submucosal fluid.

A submucosal injection solution is a special protective agent that lift dysplastic lesions in the digestive tract for endoscopic mucosal resection (EMR) or ESD, facilitating lesion removal, and acting as a barrier to prevent perforation and thermal injury. Since normal saline was first studied in EMR, many kinds of submucosal solutions, including 5% dextrose water, glycerol, hydroxypropyl methylcellulose, sodium alginate, sodium hyaluronate, and poloxamer have been studied [5, 6]. However, these solutions have limitations in terms of viscosity, price, and safety.

To overcome these shortcomings, numerous attempts have been made to adapt hydrogel as a submucosal injection solution [7–12]. Water-soluble polymer hydrogels possess hydrophilic and polymeric networks that construct three-dimensional crosslinks through physical or chemical bonds [13]. Hydrogels have long been recognized to be associated with a high proportion of water components and high physicochemical similarity with the extracellular matrix, enabling them to be an excellent biocompatible product. Therefore, they have been used extensively for biomedical applications, such as wound healing and lenses [14].

Chitosan is one of the most frequently utilized hydrogel materials and is the only natural cationic polymer that contains an amine group [15]. It has generally been accepted that chitosan has excellent biocompatibility, low toxicity, and immunostimulatory activities, mediated by its deacetylation of chitin, a natural polysaccharide [16]. Although there are various methods for manufacturing chitosan-based hydrogels, recent investigations have reported the introduction of a thermosensitive hydrogel using beta-glycerophosphate [17, 18]. When β-glycerophosphate, a natural organic compound in the human body, is mixed with chitosan, a newly developed hydrogen bond between chitosan and β-glycerophosphate transforms chitosan into a hydrogel by reducing electro-repulsion at a normal physiological temperature (37°C) [19, 20].

To the best of our knowledge, this is the first study to evaluate a thermosensitive chitosan hydrogel for ESD. The goal of this research was to determine whether the efficacy and safety of the hydrogel was superior to that of the existing outstanding submucosal fluid in the gastrointestinal tract.

## Materials & methods

### Animal and experiment preparation

**Fig 1:**
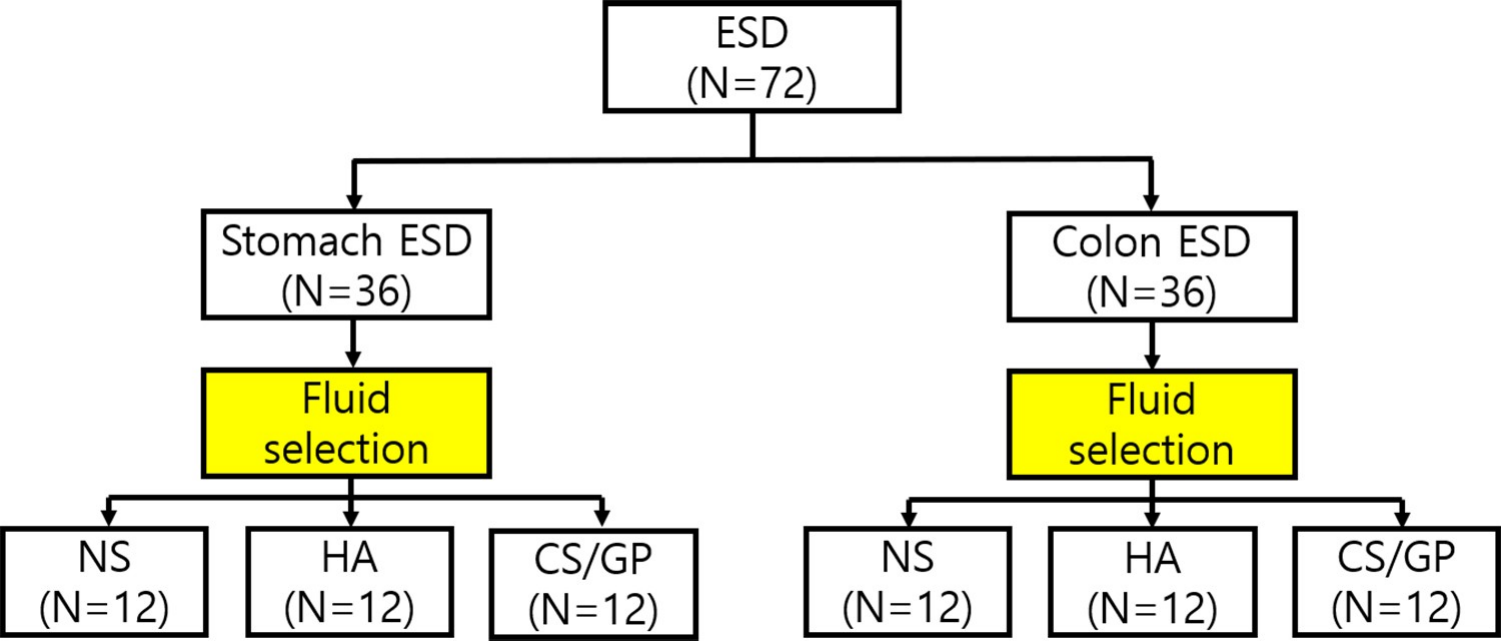
Schematic flow chart of experiments.

To perform ESD, healthy female YLD crossbred pigs (Yorkshire × Landrace × Duroc, 12 wk, mean weight 39.3 kg, XP-bio Inc., Gyeonggi-do, Korea) were acclimated to the animal laboratory (one pig/cage, 50% relative humidity, 23°C temperature, 12-h light/dark cycle) for 7 days and fasted for 2 days before the experiment. Bowel preparations were conducted using intestinal cleansing agents (Colyte Powder, Taejoon Pharm Co., Korea). All the pigs were included and premedicated with an intramuscular (IM) injection of atropine (0.02~0.04mg/kg) 1 h before the experiment and anesthetized using azaperone (2~8mg/kg, IM), xylazine (1mg/kg, IM) and alfaxalone (4mg/kg, IM). Thereafter, an endotracheal tube (6.5 Fr, Henan Tuoren Medical Device Co., Henan, China) with a balloon was inserted. The pigs were ventilated in a volume-controlled mode and anesthesia was maintained by isoflurane (1.5–2.0%) with an inspiratory fraction of oxygen of 0.5 to 0.7 (oxygen enriched air) and a tidal volume of 10 to 15ml/kg for 13 breaths/min. Respiratory settings were changed to achieve end-tidal carbon dioxide levels of 30–40 mmHg, more than 60 pulses/min, and saturated oxygen levels of 100%. The depth of anesthesia was assessed using jaw tone and the rigidity of the mandibular muscles. The core body temperature was measured in the esophagus and rectum using a thermometer before the procedure. Following anesthesia, ESD was performed sequentially in the stomach and colon (Fig 1) with vital signs and electrocardiography continuously monitored. There were no adverse events during anesthesia, and Hartmann’s dex solution (5ml/kg/hr) was administered during the experiment. Finally, the experimented pigs were euthanized by means of potassium chloride (2 mmol/kg) injected intravenously after 7 days.

A total of 72 endoscopic submucosal dissections (ESD) was performed on 12 pigs, 36 times in stomach, 36 times in colon. In one pig, six ESDs were performed either with three different solutions, normal saline (NS), hyaluronic acid (HA) and chitosan/β-glycerophosphate (CS/GP) in the stomach or colon.

### Ethics statement

The Institutional Animal Care and Use Committee of Korea University College of Medicine approved the study protocol (IACUC number: KOREA-2020-0127). All procedures on live animals were also performed according to the Animal Research: Reporting of In Vivo Experiments (ARRIVE) guidelines. All procedures were performed under general anesthesia, and all efforts were made to minimize suffering.

### Injection fluid preparation

Three solutions were prepared for submucosal injection, of which 0.9% normal saline (NS, JW Pharmaceutical, Seoul, Korea) was allocated to the control group, while 0.4% hyaluronic acid (HA, Blue Eye 1, The Standard Co. Ltd., Gyeonggi-do, Korea) and chitosan/β-glycerophosphate hydrogels (CS/GP, Blue Eye 2, The Standard. Co. Ltd., Gyeonggi-do, Korea) were used in the experimental groups. A 28% (w/v) concentrated liquid CS/GP hydrogel precursor solution was fabricated by mixing 56% (w/v) β-glycerophosphate disodium salt hydrate (Sigma Aldrich, Germany) with 1% (w/v) chitosan (HEPPE MEDICAL Chitosan GmbH, Germany) dissolved in 1% (w/v) lactic acid (Sigma Aldrich, Germany). The viscosity began to rise at 36.5°C for 7 min, and the solution turned into a complete solid after 28 min. In addition, chitosan changed into a solid phase with a sharp increase in viscosity at 38~39°C.

### ESD procedure

**Fig 2:**
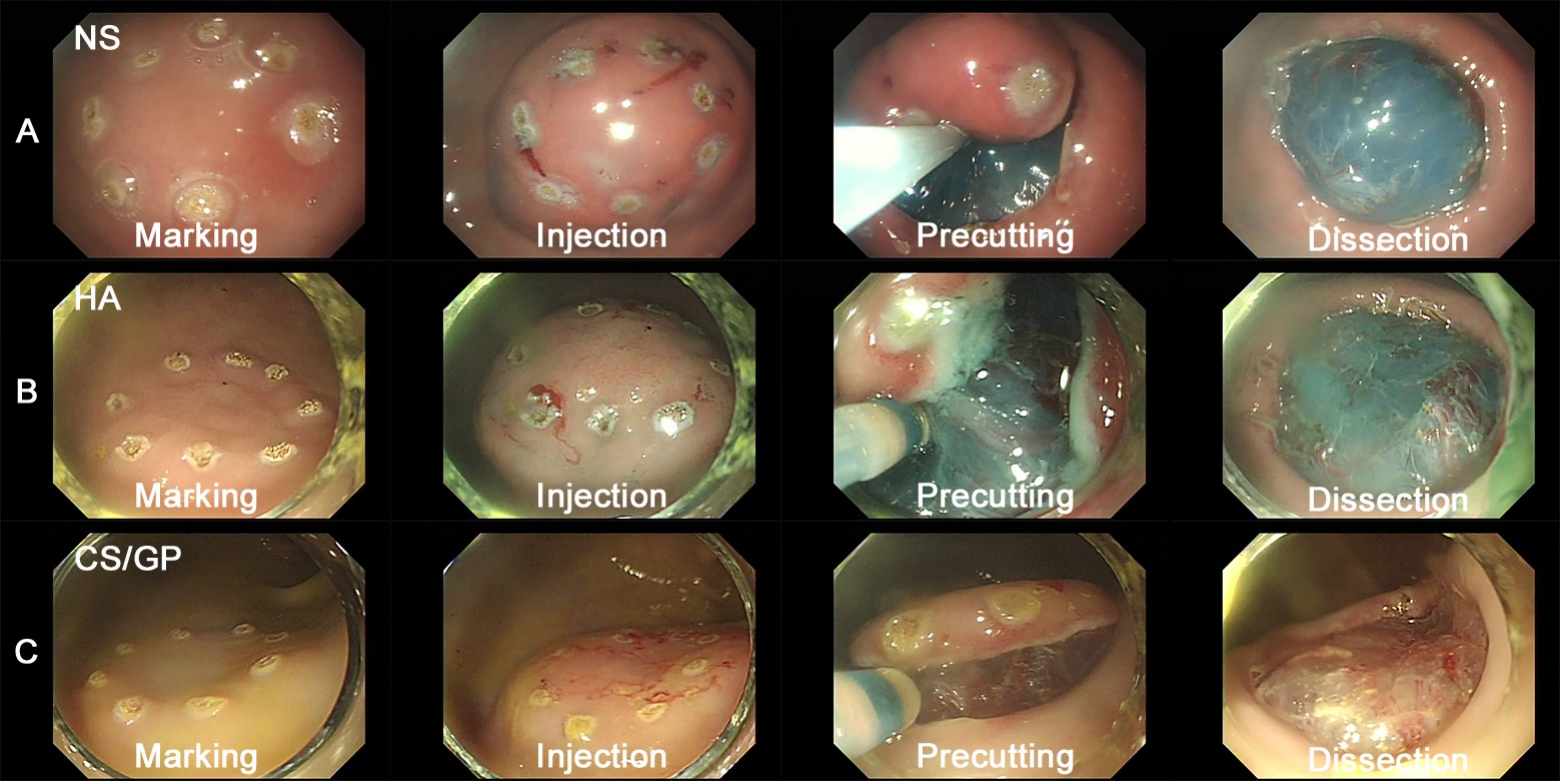
Endoscopic view of submucosal dissection procedure according to solution. (A) 0.9% normal saline (B) 0.4% hyaluronic acid (C) chitosan/β-glycerophosphate (first row, marking; second row, injection; third row, precutting; fourth row, dissection).

One pig received an ESD procedure by an experienced endoscopist (with experience of >300 cases of ESD) in the antrum, body of the stomach, and 20, 40, and 60 cm from the anal verge consecutively for a resected specimen size of approximately 2 cm×2 cm (Table 1). An assistant endoscopist, who allocated and recorded the procedure-related parameters, and a procedure endoscopist was completely blind to the solution type. After determining the location, submucosal fluid was administered at the lesion for elevation using an injector (NM-600L-0423, Stomach, Tokyo, Japan; Colon: NM-610U-0425, Olympus, Japan). A dual knife J (Stomach: KD-655L, Olympus, Japan; Colon: KD-655U, Olympus, Japan) was used to incise the peripheral mucosa of the marking site and to dissect the submucosa. An electrosurgical unit (VIO 300D; ERBE Elektromedizin, Tübingen, Germany) was introduced to the ESD procedure. The detailed electrical specifications are as follows: Endocut I for the stomach and colon (effect 2, duration 2, interval 2) and swift-coagulation mode (stomach: effect 4, maximum watt 50, colon: effect 2, watt 40). Following dissection, endoscopist-manipulated hemostatic forceps (Stomach: FD-410LR, Olympus, Japan, Colon: FD-411UR, Olympus, Japan) were used when hemostasis was required, using a soft-coagulation mode (stomach and colon: effect 6, maximum watt 60) (Fig 2). The removed flap was thinly spread on a cork board, fixed, and photographed, and the area was calculated using the Image J program (Version 1.8, National Institutes of Health, USA).

**Table 1:** Random allocation of submucosal fluids according to location.

**Stomach**	Pig 1	Pig 2	Pig 3	Pig 4	Pig 5	Pig 6	Pig 7	Pig 8	Pig 9	Pig 10	Pig 11	Pig 12
Antrum 1	NS	CS/GP	HA	NS	CS/GP	HA	NS	CS/GP	HA	NS	CS/GP	HA
Antrum 2	HA	NS	CS/GP	HA	NS	CS/GP	HA	NS	CS/GP	HA	NS	CS/GP
Body	CS/GP	HA	NS	CS/GP	HA	NS	CS/GP	HA	NS	CS/GP	HA	NS
**Colon**	Pig 1	Pig 2	Pig 3	Pig 4	Pig 5	Pig 6	Pig 7	Pig 8	Pig 9	Pig 10	Pig 11	Pig 12
AV 20cm	NS	CS/GP	HA	NS	CS/GP	HA	NS	CS/GP	HA	NS	CS/GP	HA
AV 40cm	HA	NS	CS/GP	HA	NS	CS/GP	HA	NS	CS/GP	HA	NS	CS/GP
AV 60cm	CS/GP	HA	NS	CS/GP	HA	NS	CS/GP	HA	NS	CS/GP	HA	NS

NS, 0.9% normal saline; HA, 0.4% hyaluronic acid; CS/GP, chitosan/β-glycerophosphate; AV; anal verge; Antrum 1, Antrum/Greater curvature; Antrum 2, Antrum/Anterior wall.

### Parameter definitions and outcomes

Procedure time was defined as the time from marking to hemostasis, and the dissection time was defined as the time from the start to the end of the dissection. The total volume injection, as the fluid amount used during ESD, was one of the primary endpoints. This was divided into the initial injection and additional injection volumes as secondary outcomes.


totalvolume=initialvolume+additionalvolume

Specifically, the total injected volume was expressed in terms of the initial injected volume for lifting the mucosa, together with the additional injected volume, which was the extra volume injected during the procedure.

Another primary endpoint, dissection speed (mm^2^/min), was converted into a variable related to fluid volume, which was multiplied by the fluid power and fluid consumption rate, each of which was regarded as a secondary endpoint.


$$dis\sec ion\;speed(\frac{mm^{2}}{\min})=fluid\;power(\frac{mm^{2}}{mL})\times fluid\;consumption\;rate(\frac{mL}{\min})$$

Fluid power refers to the dissection area per 1 mL of fluid volume, and the fluid consumption rate was defined as the volume of fluid consumed for 1 min. Other secondary outcomes included procedure time, bleeding, perforation, and en bloc resection.

### Histopathological assessment

The ulcer created after ESD was obtained through necropsy one week later, and its histology was analyzed for each solution tested through hematoxylin and eosin staining. Resected specimens from the stomach and colon were sliced into 3-mm sections, fixed, and observed using an optical microscope (BX51, Olympus, Japan) by a pathologist.

### Statistics

#### Sample size calculation

A preliminary study was conducted on three pigs to calculate an adequate minimal sample size (S1 Table). An effect size of 0.627 was calculated using the ANOVA (analysis of variance) in the G-power program (version 3.1.9.7.). Based on the acquired effect size, the required number of experiments obtained through a significance level of 0.05 and power of 0.8 was estimated to be approximately 12 times per solution, considering a dropout rate of 15% due to complications such as infection and perforation.

#### Statistical analyses

Primary and secondary outcomes, satisfying the normality test as continuous variables, were analyzed using the ANOVA and Tukey’s post hoc tests, and related data were expressed in terms of the mean and standard deviation. Categorical variables were presented as proportions (%) and analyzed using the χ2 and Fisher’s exact tests. Variables that did not meet normality were analyzed using non-parametric methods such as the Kruskal–Wallis test with the Mann–Whitney test and post-hoc Bonferroni correction. Statistical significance was considered as p<0.05, and p<0.0167 was considered significant with the Bonferroni correction. All statistical analyses were performed using the SPSS program (ver. 24; IBM Corp., Armonk, NY, USA).

## Results

### Study population

A total of 72 ESD procedures were performed on 12 pigs, of which 36 were performed in the stomach. The study was designed for a single pig undergoing ESD six times in total, three in stomach with three different submucosal fluids administered once in each lesion of interest, and the same protocol applies to the colon. No animals were excluded during the study protocol.

### Stomach ESD efficacy

As shown in Table 2, there was a significant difference in the total injected volume comprising the initial and additionally injected volumes among the three groups. HA and CS/GP solutions required significantly less volume than NS, suggesting a higher volume of NS was required to complete ESD. There was no difference between HA and CS/GP groups. The most frequent number of injections was two for all solutions, and CS/GP showed the highest rate of completion with only one injection.

**Table 2:** Comparative gastric endoscopic submucosal dissection-related parameters between solutions.

Stomach	NS (n = 12)	HA (n = 12)	CS/GP (n = 12)	p-value
Total volume (mL)	16.09±3.27	11.17±2.32	9.44±2.33	<0.001^a^
Initial volume	10.99±2.22	8.63±2.20	7.95±1.26	0.001^a^
Additional volume	5.10±2.28	2.54±0.62	1.49±1.32	<0.001^a^
Injection number				0.332^b^
1	0/12 (0.0%)	2/12 (16.7%)	3/12 (25.0%)	
2	11/12 (91.7%)	10/12 (83.3%)	9/12 (75.0%)	
3	1/12 (8.3%)	0/12 (0.0%)	0/12 (0.0%)	
Procedure time (min)	14.92±4.44	15±2.98	14.08±2.87	0.780^a^
Dissection time (min)	6.92±2.81	6.58±2.19	6.42±2.27	0.878^a^
Dissection speed (cm^2^/min)	88.99±28.53	93.62±39.36	92.40±41.79	0.951^a^
Power (cm^2^/mL)	35.70±9.00	47.74±7.90	57.48±20.77	0.002^a^
Consumption rate (mL/min)	2.59±0.86	1.80±0.82	1.62±0.65	0.011^a^
ESD area (mm^2^)	566.07±161.46	532.49±128.68	533.65±202.80	0.856^a^
ESD complication				
Bleeding	2/12 (0%)	1/12 (0%)	2/12 (0%)	1.000^b^
Perforation	0/12 (0%)	0/12 (0%)	0/12 (0%)	1.000^b^
En-bloc resection	100%	100%	100%	1.000^b^

ESD, endoscopic submucosal dissection; NS, 0.9% normal saline; HA, 0.4% hyaluronic acid; CS/GP, chitosan/β-glycerophosphate.

^a^, ANOVA (analysis of variance).

^b^, Fisher’s exact test.

All values are significant at p < 0.05 level.

Normality test was significant at p > 0.05 level.

No statistically significant difference was observed in the procedure and dissection time between the solutions. However, the power and consumption rates, which are components of dissection speed, proved to be statistically significant between NS and CS/GP. This indicates that CS/GP, compared to NS, has a high power to separate larger mucosal areas per 1 mL, with lower absorption rates in the submucosa (Table 3).

**Table 3:** Post hoc analysis of endoscopic submucosal dissection parameters.

	NS	NS	HA
	vs	vs	vs
	HA	CS/GP	CS/GP
**Stomach**			
Total volume^a^	<0.001	<0.001	0.269
Initial volume^a^	0.014	0.002	0.674
Additional volume^a^	<0.001	<0.001	0.241
Fluid Power^a^	0.099	0.001	0.211
Consumption rate^a^	0.049	0.013	0.838
**Colon**			
Total volume^a^	0.002	0.003	0.991
Initial volume^a^	0.116	0.286	0.865
Additional volume^b^	0.035*	0.01*	0.836*
Fluid Power^a^	0.449	0.506	0.995
Consumption rate^a^	0.299	0.291	1.000

NS, 0.9% normal saline; HA, 0.4% hyaluronic acid; CS/GP, chitosan/β-glycerophosphate.

^a^, Tukey post-hoc.

^b^, Mann-Whitney post-hoc.

*, p-value < 0.167 in accordance with Bonferroni correction.

### Colon ESD efficacy

The results of colon ESD are recorded in Table 4. The HA and CS/GP solution significantly reduced the use of submucosal fluid amounts to less than that of the NS in the total injection volume. In the stomach, the initial injection volumes between solutions did not differ. With regard to additional volumes injected, the experimental groups, HA and CS/GP, were clearly different compared to those of the control groups (NS vs. HA; p = 0.035, NS vs. CS/GP; p = 0.01) (Table 3). The solution that could accomplish the procedure with one injection was most likely to be the HA and CS/GP solution, while NS required the higher rate of necessitated more than two injections most of the time during the procedure. The dissection speed with its subcomponent fluid power and consumption rate showed no statistical significance.

**Table 4:** Comparative colonic endoscopic submucosal dissection-related parameters between solutions.

Colon	NS (n = 12)	HA (n = 12)	CS/GP (n = 12)	p-value
Total volume (mL)	9.17±1.80	6.67±1.50	6.75±1.57	0.001^a^
Initial volume	7.17±1.85	5.83±1.34	6.17±1.54	0.118^a^
Additional volume^a^	2.00±1.19	0.83±1.40	0.58±0.97	0.002^b^
Injection number				0.060^c^
1	3/12 (25.0%)	8/12 (66.7%)	8/12 (66.7%)	
2	9/12 (75.0%)	3/12 (25.0%)	4/12 (33.3%)	
3	0/12 (0.0%)	1/12 (8.3%)	0/12 (0.0%)	
Procedure time (min)	11.33±1.92	10.17±2.37	9.67±2.53	0.203^a^
Dissection time (min)	6.83±1.70	6.17±2.12	6.08±1.78	0.568^a^
Dissection speed (cm^2^/min)	59.67±26.02	59.02±27.55	55.02±17.59	0.878^a^
Power (cm^2^/mL)	41.89±16.15	53.07±23.32	52.17±26.46	0.407^a^
Consumption rate (mL/min)	1.41±0.43	1.18±0.40	1.18±0.30	0.230^a^
ESD area (mm^2^)	373.36±131.30	338.98±139.84	341.86±164.64	0.816^a^
ESD complication				
Bleeding	1/12 (8.3%)	0/12 (0.0%)	2/12 (16.7%)	1.000^c^
Perforation	0/12 (0.0%)	0/12 (0.0%)	0/12 (0.0%)	1.000^c^
En-bloc resection	100%	100%	100%	1.000^c^

ESD, endoscopic submucosal dissection; NS, 0.9% normal saline; HA, 0.4% hyaluronic acid; CS/GP, chitosan/β-glycerophosphate.

All values are significant at p < 0.05 level.

Normality test was significant at p > 0.05 level.

^a^, ANOVA (analysis of variance).

^b^, Kruskal-Wallis test.

^c^, Fisher’s exact test.

#### Histopathology

**Fig 3:**
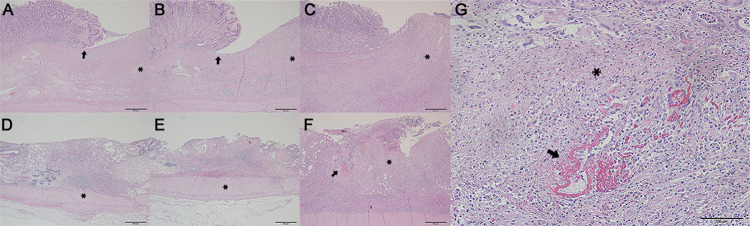
Histologic representation of endoscopic submucosal dissection (ESD) induced ulcer after 7 days. The first and second row indicate gastric and colonic ulcers after ESD, respectively. Administered by (A) normal saline and (B) 0.4% hyaluronic acid revealing a microscopically sharply demarcated ulcer (arrow) without hemorrhage (asterisk), and by (C) chitosan/β-glycerophosphate, presenting a loss of the mucosal layer with a hypertrophied submucosal layer (asterisk) at the ulcer base. Hematoxylin-Eosin (H & E) stain; x40. The colonic ulcer injected by (D) normal saline, (E) 0.4% hyaluronic acid showing a smooth margin without hemorrhage and perforation (asterisk), and (F) a chitosan/β-glycerophosphate ulcer presenting with a thickened submucosal layer (asterisk), and focal lysis (arrow) with granulation tissue, H & E; x40, (G) The gastric tissue administered with chitosan/β-glycerophosphate solution exhibiting focal cell lysis (asterisk) and fibrinolysis (arrow), which is indicative of minimal hemorrhaging (strong pink). H & E; x200.

Histology showed structural destruction of epithelial tissues with a loss of cellular components. Granulation tissue and the infiltration of inflammatory cells were confirmed 24-h later in the ulcer bed from the ESD site for all three solutions. Overall, there was no significant difference in terms of the lesions’ gross inspection and the degree and extent of their ulceration between the three solutions It was also noted that the muscularis propria layer revealed no inflammatory reaction. (Fig 3A–3F). However, submucosal thickening (Fig 3C and 3F) and cell lysis with peripheral focal fibrinolysis was observed with local hemorrhages beneath the ulcer bed in CS/GP groups (Fig 3G).

## Discussion

The majority of tumors arising from the gastrointestinal tract (GIT) occurs as a result of disorganization of the mucosal architecture [21]. In particular, the epithelium, which distinguishes the inner and outer membranes of the body, is a tissue that initiates gastrointestinal tract dysplasia [22]. Therefore, endoscopic treatment that can identify a dysplastic epithelium at an early stage and selectively isolate it from normal tissue is crucial. EMR and ESD represent a standard, advanced endoscopic techniques that can eliminate the early undesirable tissue of the GIT by predominantly empolying submucosal injection fluid.

The first objective of this study was to assess the efficacy and safety of a CS/GP thermosensitive system for GIT. In particular, this study demonstrates the ESD procedure results using CS/GP submucosal solutions and compare them to those of HA. Our results indicated that CS/GP is much more efficient than NS with regard to the amount of fluid required to complete the ESD (total injected volume, stomach; NS vs. CS, p<0.005).

The outcomes of this study correspond with those of an earlier study that reported the binary hydrogel system to be significantly superior to NS in fluid volume and wound healing [23]. Data from experimental animals showed that the thermosensitive binary hydrogel system (TBHS), comprising poloxamer 407 and 188, required less total injected volume than NS (22.13 mL vs. 6.63 mL, p<0.001). The report speculated that one of the reasons why TBHS does not spread to the surrounding submucosa was because of its ability to be converted into the phase of high viscosity once it reaches above the gelation temperature.

Although this study was designed to dissect similar specimen areas, the initial volumes necessary to make an identically sized submucosal fluid cushion were significantly different in the stomach. The initial injected volumes required in the colon, on the other hand, produced fairly even specimen areas (stomach, p = 0.001 vs. colon, p = 0.118). It is presumed that the differences in initial volumes between the stomach and colon are primarily due to the different ESD areas and the thickness of the submucosal layers, resulting in different injection amounts required to elevate the cushion to an appropriate height [24].

It was originally expected the number of injections of CS/GP or HA to be less and statistically different from NS. Based on the results, however, there was no significant difference. Nevertheless, the proportion of cases with more than two injections was 75% in normal saline group in the colon, whereas 25% in HA and 33.3% in CS/GP groups. Out results are consistent with prior study of Eleview (EMR: 1.5±1 vs 2.1±1.8, p = ns, ESD: 9.4±12.1vs 12.1±0.4, p = ns) [25], but contradict those of the TBHS (p = 0.007) (23).

The ESD procedure in this study satisfied the skilled ESD performance criteria, attaining dissection speed >9 cm^2^/h, adverse events rate <5%, and en bloc resection rate >90% [26]. ESD speed showed a minimal disparity among different types of solutions used, which could be due to intrinsic selection bias, as endoscopists may have preferred the locations with relatively easier access. Another explanation can be attributed to the endoscopists ability itself. It has been reported that dissection speed commonly does not vary when endoscopists, who have obtained proficiency in ESD, are involved [23]. Dissection speed was, therefore, broken down into two volume parameters, fluid power and fluid consumption rate, of which the values varied depending on the types of solutions used. Given the same dissection speed, the larger the fluid power, the smaller the fluid consumption rate, which implies the corresponding submucosal solution has exhibited a better performance in terms of “volume-efficacy.”

Concerning gastric ESD, there were significant differences in fluid power and consumption rates (Table 2), whereas colorectal ESD showed no differences (Table 4). The results of colon ESD indicated that HA and CS/GP have higher fluid power than NS (NS; 41.89±16.15 vs. BE; 53.07±23.32 vs. CS; 52.17±26.46), and vice versa in consumption rates (NS; 1.41±0.43 vs. BE; 1.18±0.40 vs. CS; 1.18±0.30). It can thus be postulated that statistical significance in the colon will be evident as the sample size increases.

The strength of this study was in that the CS/GP system was compared to HA to validate the efficacy of ESD, implemented both in stomach and colon simultaneously. The photocrosslinkable chitosan derivative in the previous study had to be transformed into a chitosan hydrogel by UV irradiation [8, 27], which has curtailed the simplicity of the process with the need for time and extra equipment to perform UV irradiation. The major concern unresolved by the previous studies was whether the chitosan solution could change naturally by itself and untangle during the hydrogel process. Our study design has amended such drawbacks by physiologically transforming chitosan into a hydrogel with increased viscosity which enabled fluid cushions to sustain for longer durations without additional steps with its low cost (4$/mL).

This experiment has some limitations, mostly stemming from the small sample size. In addition, the involved endoscopist may have displayed involuntary bias. With chitosan being clear and yellowish, and HA and NS being blue, their colors may have given a clue to the practitioner which type they are dealing with at the time of each procedure. Therefore, a performance bias could have been elicited to adjust the injection capacity or speed between solutions. Second, the average temperatures measured in the stomach and rectum were 37.5°C and 37.3°C, respectively, which were slightly lower than the average pig’s initial body temperature (38–40°C). This is presumed to be as a result of anesthesia and enema. The longer the anesthesia gets prolonged, the lower the body temperature becomes. Furthermore, the enema fluid remaining in the colon may have influenced the rectal temperature measurement [28]. Therefore, it is postulated that the temperature effect of the ESD in colon could have been less prominent compared to that in stomach, since the colon procedure was initiated after the completion of the stomach ESD. Third, there was no histologic difference in the artificial ulcer after ESD, although the CS/GP solution showed thickening of the submucosal layer at the ulcer bed in addition to a focal hemorrhage. It is speculated that these findings could be attributed to excessive extracellular matrices with relatively high osmolarity [29]. Therefore, it appears that a slight modification, such as a concentration of chitosan derivatives, might be necessary for the creation of favorable submucosal fluids.

## Conclusion

This study demonstrated that the amount of CS/GP solution is required much less in ESD procedure than that of normal saline, while the amount of volume was not distinctly different when hyaluronic acid was used. The findings in this animal experiment provide strong in vivo evidence of the CS/GP system as a candidate for another effective submucosal injection fluid. Future studies will be required to focus on pre-clinical studies and CS/GP with long-term histopathologic analysis related to tissue recovery after ESD.

## Supporting information

S1 Checklist The ARRIVE guidelines 2.0: Author checklist. (PDF)

S1 Table Minimal raw dataset of injected volume profile of solutions administered in three pigs. (DOCX)
