# Supplementary material for: Efficacy and safety of a thermosensitive hydrogel for endoscopic submucosal dissection: An in vivo swine study
Source: PLoS One. 2021 Dec 9;16(12):e0260458. doi: 10.1371/journal.pone.0260458 (PMC8659419; doi:10.1371/journal.pone.0260458)
Supplement: S1 Table — (DOCX) [file pone.0260458.s002.docx]

S1 Table. Minimal raw dataset of injected volume profile of solutions administered in three pigs.

| Pig number | Solution | Initial volume (ml) | Additional volume (ml) | Total volume (ml) | Injection number |
| --- | --- | --- | --- | --- | --- |
| 1 | Normal saline | 16 | 8 | 24 | 2 |
| 1 | Hyaluronic acid | 13.3 | 8.3 | 21.6 | 3 |
| 1 | chitosan/β-glycerophosphate | 14 | 8 | 22 | 2 |
| 2 | Normal saline | 9.5 | 6.5 | 16 | 2 |
| 2 | Hyaluronic acid | 9 | 3.5 | 12.5 | 2 |
| 2 | chitosan/β-glycerophosphate | 9.5 | 4 | 13.5 | 2 |
| 3 | Normal saline | 11.5 | 8.5 | 20 | 3 |
| 3 | Hyaluronic acid | 7 | 3 | 10 | 2 |
| 3 | chitosan/β-glycerophosphate | 8.5 | 2 | 10.5 | 2 |
